# Dengue Virus IgM Serotyping by ELISA with Recombinant Mutant Envelope Proteins

**DOI:** 10.3201/eid2501.180605

**Published:** 2019-01

**Authors:** Alexandra Rockstroh, Luisa Barzon, Widuranga Kumbukgolla, Hoang Xuan Su, Erley Lizarazo, Maria Fernanda Vincenti-Gonzalez, Adriana Tami, Alice M.M. Ornelas, Renato Santana Aguiar, Daniel Cadar, Jonas Schmidt-Chanasit, Sebastian Ulbert

**Affiliations:** Fraunhofer Institute for Cell Therapy and Immunology, Leipzig, Germany (A. Rockstroh, S. Ulbert);; University of Padova, Padova, Italy (L. Barzon);; Rajarata University of Sri Lanka, Mihinthale, Sri Lanka (W. Kumbukgolla);; Vietnam Military Medical University, Hanoi, Vietnam (H.X. Su);; University Medical Center Groningen, Groningen, the Netherlands (E. Lizarazo, M.F. Vincenti-Gonzalez, A. Tami);; Federal University of Rio de Janeiro, Rio de Janeiro, Brazil (A.M.M. Ornelas, R.S. Aguiar);; Bernhard Nocht Institute for Tropical Medicine, Hamburg, Germany (D. Cadar, J. Schmidt-Chanasit);; German Centre for Infection Research, Hamburg (J. Schmidt-Chanasit)

**Keywords:** dengue, serotypes, diagnosis, diagnostics, ELISA, IgM, envelope, viruses, vector-borne infections, methods, dengue virus, Germany, Italy, serologic test, Brazil, Venezuela, Sri Lanka, Vietnam, travelers, flavivirus, fusion loop, cross-reactivity

## Abstract

We developed an IgM-based ELISA that identifies the dengue virus serotype of recent infections. Dominant serotypes were detectable in 91.1% of samples from travelers and 86.5% of samples from residents of endemic regions; 97.1% corresponded to the serotype identified by PCR. This ELISA enables more accurate reporting of epidemiologic findings.

Dengue virus (DENV) is an arthropodborne flavivirus that is endemic in tropical and subtropical regions, causing hundreds of millions of infections annually (*1*). It is subdivided into 4 serotypes, DENV-1–4. After infection, patients have lifelong immunity against the homologous serotype but remain susceptible to infections with the others (*2*). Such secondary infections have been shown to be a risk factor for severe dengue with life-threatening clinical manifestations, including dengue hemorrhagic fever or dengue shock syndrome (*3*). Thus, monitoring the serotype is essential for outbreak management, epidemiologic studies, and patient care. Analyses are often performed by using direct virus detection methods, such as PCR and nonstructural protein 1 (NS1) antigen capture (*4*). Despite the high specificities of these assays, their main disadvantages include a rather small diagnostic window for detection and, for NS1 antigen capture tests, low sensitivities during secondary DENV infections (*5*). Identification of the infecting serotype by serologic methods is hampered by the cross-reactivity of antibodies elicited by the immune response against flaviviruses (*6*). Previous reports have shown that the insertion of mutations in the conserved fusion loop domain of flavivirus envelope proteins reduces this cross-reactivity in diagnostic testing (*7*–*9*). Using this method, we developed a DENV-specific ELISA capable of differentiating DENV from other clinically relevant flaviviruses, such as Zika virus, West Nile virus, and tick-borne encephalitis virus (*10*). In this study, we evaluated the potential of this technique to distinguish the 4 DENV serotypes during the acute phase of infection on the basis of IgM detection.

## The Study

We acquired DENV PCR–confirmed and, thereby, serotype-classified serum samples that we divided into 2 groups: those from returning travelers with residence in Italy or Germany (n = 45), collected from patients 5–60 days after symptom onset during 2013–2016; and those from persons residing in the DENV-endemic countries of Sri Lanka (n = 43), Vietnam (n = 24), Venezuela (n = 5), and Brazil (n = 2), collected from patients 2–8 days after symptom onset during 2013–2018. We also had a set of 14 DENV PCR–negative but NS1-positive (PLATEILA DENGUE NS1 AG; Bio-Rad, Hercules, CA, USA) serum samples from patients in Vietnam that were collected during the same outbreak as the other patients from Vietnam with DENV PCR–positive test results. Ethics approvals were obtained from the respective local authorities for all samples.

We first tested all serum samples with a DENV-specific ELISA that used 4 recombinant DENV envelope proteins (1 per serotype) containing 4 point mutations in and near the conserved fusion loop (called Equad proteins) (*9*). All samples were positive for DENV IgM (S. Ulbert, unpub. data). This Equad-based ELISA was previously shown to be capable of discriminating DENV from other flaviviruses (*9*,*10*). We took the Equad antigens from this ELISA and created a DENV serotyping ELISA. In brief, we titrated serum samples (1:100–1:12,800 in serial 2-fold dilutions) in duplicate on plates coated with DENV-1–4 Equad proteins (Appendix) (*10*). We defined the endpoint titer for every serotype as the last dilution presenting a signal above the cutoff (Appendix Table), which was calculated as the mean plus 2 times the SD of 15 flavivirus-negative control serum samples (diluted 1:100) acquired from Padova University Hospital, Padova, Italy.

**Figure 1:**
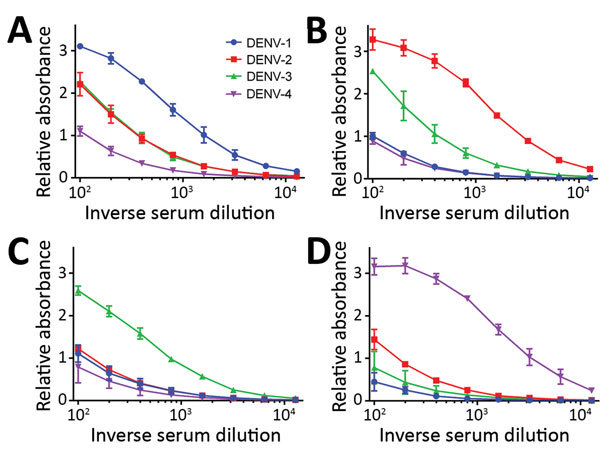
DENV IgM ELISA titers, by serotype, for DENV PCR–positive serum samples from travelers returning to Germany or Italy, 2013–2016. A) DENV-1; B) DENV-2; C) DENV-3; D) DENV-4. Data lines indicate average titers; error bars indicate SDs. The antigens in this ELISA were Equad proteins (i.e., envelope protein from each DENV serotype with 4 amino acid changes T76R, Q77E, W101R, and L107R). In these examples, the highest endpoint titers corresponded to the DENV serotype identified by PCR analysis. DENV, dengue virus.

Results showed 1 dominant serotype (Figure 1) for 91.1% of serum samples from returning travelers and 86.5% of serum samples from residents of DENV-endemic countries (Appendix Figures 1, 2). Compared with samples from Vietnam, 15% fewer samples from Sri Lanka had a dominant serotype (Table 1). Serum samples from patients in Sri Lanka cross-reacted only between serotypes 1 and 2 (Appendix Figure 2); however, these samples were collected at a time when the dominant serotype in circulation was switching from DENV-1 to DENV-2 after a DENV-1 outbreak in early 2016 (W. Kumbukgolla, unpub. data). Therefore, this result could be explained by preexisting IgM or, alternatively, by co-infections. However, co-infections were not evident by PCR.

**Table 1:** Dominant serotype determined by DENV serotype–specific IgM ELISA and ELISA specificity, by cohort and serotype, 2013–2018*

Cohort, DENV serotype†	No. samples	Dominant serotype, no. (%) samples	Serotype specificity‡	
			No. samples	% Samples (95% CI)
Returning travelers				
DENV-1	16	14 (87.50)	14	87.50 (61.65–98.45)
DENV-2	11	10 (90.91)	8	72.73 (39.03–93.98)
DENV-3	10	9 (90)	9	90.00 (55.50–99.75)
DENV-4	8	8 (100.00)	7	87.50 (47.35–99.68)
All serotypes	45	41 (91.11)	38	84.44 (70.54–93.51)
Residents in DENV-endemic countries				
Sri Lanka				
DENV-1	23	16 (69.57)	16	69.57 (47.08–86.79)
DENV-2	14	13 (92.86)	13	92.86 (66.13–99.82)
DENV-4	6	6 (100.00)	6	100.00 (54.07–100.00)
All serotypes	43	35 (81.40)	35	81.40 (66.60–91.61)
Vietnam				
DENV-1	20	19 (95.00)	19	95.00 (75.13–99.87)
DENV-2	4	4 (100.00)	4	100.00 (39.76–100.00)
All serotypes	24	23 (95.83)	23	95.83 (78.88–99.89)
Venezuela				
DENV-4	5	4 (80.00)	4	80.00 (23.36–99.49)
Brazil				
DENV-4	2	2 (100.00)	2	100.00 (15.81–100.00)
Total				
DENV-1	43	35 (81.40)	35	81.40 (66.60–91.61)
DENV-2	18	17 (94.44)	17	94.44 (72.71–99.86)
DENV-4	13	12 (92.31)	12	92.31 (76.55–99.81)
All serotypes	74	64 (86.49)	64	86.49 (76.55–93.32)
Total, all serotypes	119	105 (88.24)	102	85.71 (77.12–91.45)

*DENV, dengue virus. †Determined by PCR. ‡Determined by correspondence with PCR results.

Overall, for 97.1% (102/105) of samples with a dominant serotype, ELISA results corresponded with PCR results; for patients residing in endemic regions, 100% (64/64) of the sample results corresponded, and for returning travelers, 92.7% (38/41) of the sample results corresponded (Table 1). When including the samples for which no dominant serotype was detectable, the overall serotype specificity of the Equad-based ELISA was 85.7% (Table 1); specificity of the ELISA for serum samples from persons in endemic countries was 86.5% and for returning travelers 84.4%. Because of the lower number of dominant serotypes detected, the cohort from Sri Lanka displayed a lower ELISA specificity (81.4%) than did the cohort from Vietnam (95.8%). The serotype results for the PCR-negative group from Vietnam were consistent with the PCR-positive group from Vietnam: ≈80% DENV-1 and ≈20% DENV-2 (Tables 1, 2).

**Table 2:** Dominant serotype determined by DENV serotype–specific IgM ELISA among DENV PCR–negative cohort (n = 14), Vietnam, 2013–2018*

No. (%) samples with dominant serotype	DENV serotype	No. (%) samples
13 (92.86)	1	10/14 (71.43)
	2	3/14 (21.43)

*DENV, dengue virus.

**Figure 2:**
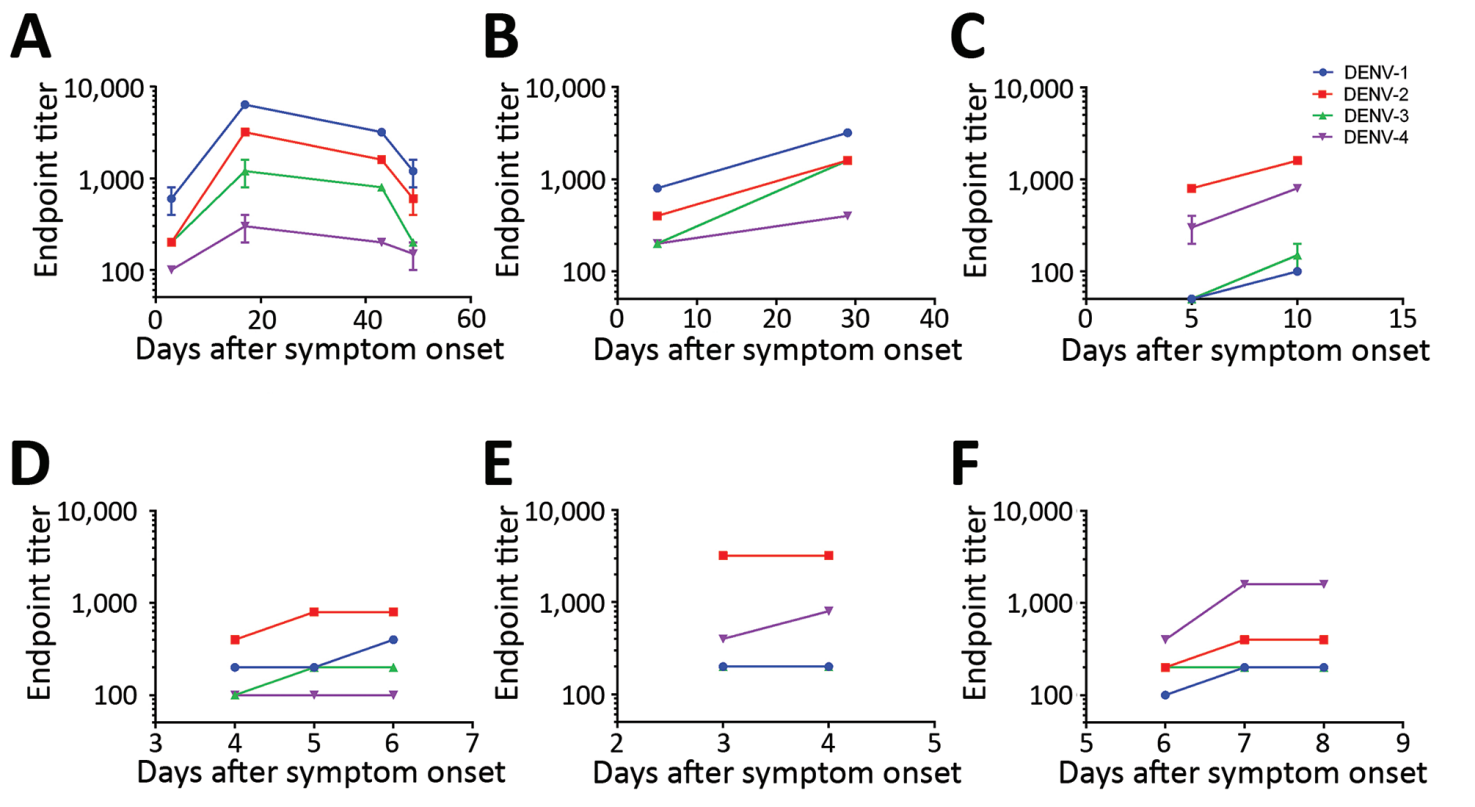
DENV IgM ELISA endpoint titers, by serotype, of paired patient serum samples acquired at 2–4 time points after symptom onset, 2013–2018. A–C) Serum samples from returning travelers with residence in Germany: DENV-1 positive (A, B); DENV-2 positive (C). D–F) Serum samples from residents of DENV-endemic country Sri Lanka: DENV-2 positive (D, E); DENV-4 positive (F). Data lines indicate average titers; error bars indicate SDs. The antigens in the ELISA were Equad proteins (i.e., envelope protein from each DENV serotype with 4 amino acid changes T76R, Q77E, W101R, and L107R). DENV, dengue virus.

Analysis of paired serum samples suggests that the results of this Equad-based ELISA are consistent over time and with different initial antibody concentrations (Figure 2). The IgM serotype initially identified as the dominant serotype remained the dominant serotype for at least 8 days (and potentially longer) after symptom onset for both returning travelers and patients residing in DENV-endemic countries, many of whom probably had secondary DENV infections (Appendix Figure 3).

## Conclusions

Early identification of the infecting DENV serotype can be a critical component of dengue diagnosis that is also essential to pathologic and epidemiologic monitoring of outbreaks. Because of its long persistence in serum, IgM is a preferred diagnostic marker, especially when viral nucleic acids and NS1 are no longer detectable. However, serologic determination of the infecting serotype is challenging. Detection of neutralizing antibodies remains the standard method for DENV identification, but interpretation is complicated by the antigenic sin phenomenon in secondary infections (*11*). The possibility of using IgM to determine the infecting serotype was attempted previously (*12*–*14*), but results were complicated by low specificities, especially for secondary DENV infections. In this study, we analyzed the serotype-specific IgM responses to DENV infections in returning travelers and residents in DENV-endemic regions using recombinant mutated envelope proteins with reduced cross-reactivity (*9*,*10*). The ELISA was able to specifically detect the infecting DENV serotype in 84.4% of travelers and 86.5% of residents in DENV-endemic regions. If a dominant serotype was detected, it corresponded to the PCR result in 100% of cases in DENV-endemic regions. The finding that some sample sets did not have a dominant IgM serotype remains to be explained. Factors such as a change in the DENV serotype during an outbreak (as seen in the Sri Lanka cohort) or differing patient exposure histories could be involved. The study included DENV-3–positive serum samples from returning travelers but not from persons residing in endemic regions. However, results obtained for the other serotypes indicate that specificities were similar for samples from returning travelers and inhabitants of endemic areas.

In summary, our results suggest that specific IgM serotyping can be achieved with an ELISA-based format when using as antigens DENV envelope proteins reduced in cross-reactivity. The test can be optimized further by, for example, varying the serum dilutions tested. By using IgM-based serologic tests, which have broad diagnostic windows (*15*), we can more accurately report epidemiologic outbreak findings.

Appendix Description of dengue virus (DENV) serotype–specific IgM ELISA, ELISA cutoff values, IgM endpoint titers of serum samples from patients traveling to and residing in DENV-endemic countries, 2013–2018, and IgM:IgG ratios of serum samples from patients residing in DENV-endemic countries, 2013–2018.

## References

[1] Bhatt S, Gething PW, Brady OJ, Messina JP, Farlow AW, Moyes CL, et al. The global distribution and burden of dengue. Nature. 2013;496:504–7. 10.1038/nature12060 23563266 PMC3651993

[2] Gubler D, Kuno G, Markoff L. Flaviviruses. In: Knipe DM, Howley PM, editors. Fields virology, vol. 1, 5th ed. Philadelphia: Lippincott Williams & Wilkins; 2007. p. 1153–252.

[3] Katzelnick LC, Gresh L, Halloran ME, Mercado JC, Kuan G, Gordon A, et al. Antibody-dependent enhancement of severe dengue disease in humans. Science. 2017;358:929–32. 10.1126/science.aan6836 29097492 PMC5858873

[4] Bosch I, de Puig H, Hiley M, Carré-Camps M, Perdomo-Celis F, Narváez CF, et al. Rapid antigen tests for dengue virus serotypes and Zika virus in patient serum. Sci Transl Med. 2017;9:eaan1589. 10.1126/scitranslmed.aan1589 28954927 PMC6612058

[5] Hang VT, Nguyet NM, Trung DT, Tricou V, Yoksan S, Dung NM, et al. Diagnostic accuracy of NS1 ELISA and lateral flow rapid tests for dengue sensitivity, specificity and relationship to viraemia and antibody responses. PLoS Negl Trop Dis. 2009;3:e360. 10.1371/journal.pntd.0000360 19156192 PMC2614471

[6] Koraka P, Zeller H, Niedrig M, Osterhaus ADME, Groen J. Reactivity of serum samples from patients with a flavivirus infection measured by immunofluorescence assay and ELISA. Microbes Infect. 2002;4:1209–15. 10.1016/S1286-4579(02)01647-7 12467761

[7] Crill WD, Chang G-JJ. Localization and characterization of flavivirus envelope glycoprotein cross-reactive epitopes. J Virol. 2004;78:13975–86. 10.1128/JVI.78.24.13975-13986.2004 15564505 PMC533943

[8] Chabierski S, Barzon L, Papa A, Niedrig M, Bramson JL, Richner JM, et al. Distinguishing West Nile virus infection using a recombinant envelope protein with mutations in the conserved fusion-loop. BMC Infect Dis. 2014;14:246. 10.1186/1471-2334-14-246 24884467 PMC4028281

[9] Rockstroh A, Barzon L, Pacenti M, Palù G, Niedrig M, Ulbert S. Recombinant envelope-proteins with mutations in the conserved fusion loop allow specific serological diagnosis of dengue-infections. PLoS Negl Trop Dis. 2015;9:e0004218. 10.1371/journal.pntd.0004218 26565964 PMC4643925

[10] Rockstroh A, Moges B, Barzon L, Sinigaglia A, Palù G, Kumbukgolla W, et al. Specific detection of dengue and Zika virus antibodies using envelope proteins with mutations in the conserved fusion loop. Emerg Microbes Infect. 2017;6:e99. 10.1038/emi.2017.87 29116222 PMC5717088

[11] Midgley CM, Bajwa-Joseph M, Vasanawathana S, Limpitikul W, Wills B, Flanagan A, et al. An in-depth analysis of original antigenic sin in dengue virus infection. J Virol. 2011;85:410–21. 10.1128/JVI.01826-10 20980526 PMC3014204

[12] Nawa M, Yamada KI, Takasaki T, Akatsuka T, Kurane I. Serotype-cross-reactive immunoglobulin M responses in dengue virus infections determined by enzyme-linked immunosorbent assay. Clin Diagn Lab Immunol. 2000;7:774–7. 10973453 10.1128/cdli.7.5.774-777.2000 PMC95954

[13] Shu P-Y, Chen L-K, Chang S-F, Su C-L, Chien L-J, Chin C, et al. Dengue virus serotyping based on envelope and membrane and nonstructural protein NS1 serotype-specific capture immunoglobulin M enzyme-linked immunosorbent assays. J Clin Microbiol. 2004;42:2489–94. 10.1128/JCM.42.6.2489-2494.2004 15184425 PMC427809

[14] Zidane N, Dussart P, Bremand L, Bedouelle H. Cross-reactivities between human IgMs and the four serotypes of dengue virus as probed with artificial homodimers of domain-III from the envelope proteins. BMC Infect Dis. 2013;13:302. 10.1186/1471-2334-13-302 23815496 PMC3701519

[15] Hunsperger EA, Yoksan S, Buchy P, Nguyen VC, Sekaran SD, Enria DA, et al. Evaluation of commercially available diagnostic tests for the detection of dengue virus NS1 antigen and anti-dengue virus IgM antibody. PLoS Negl Trop Dis. 2014;8:e3171. 10.1371/journal.pntd.0003171 25330157 PMC4199549

