# Supplementary material for: Dengue Virus IgM Serotyping by ELISA with Recombinant Mutant Envelope Proteins
Source: Emerg Infect Dis. 2019 Jan;25(1):112–5. doi: 10.3201/eid2501.180605 (PMC6302594; doi:10.3201/eid2501.180605)
Supplement: Appendix — Description of dengue virus (DENV) serotype–specific IgM ELISA, ELISA cutoff values, IgM endpoint titers of serum samples from patients traveling to and residing in DENV-endemic countries, 2013–2018, and IgM:IgG ratios of serum samples from patients residing in DENV-endemic countries, 2013–2018. [file 18-0605-Techapp-s1.pdf]

Article DOI: https://doi.org/10.3201/eid2501.180605

Dengue Virus IgM Serotyping by ELISA with Recombinant Mutant Envelope Proteins

Technical Appendix

Recombinant Proteins

Dengue virus (DENV) envelope proteins with 4 point mutations resulting in 4 amino acid changes in and near the conserved fusion loop (T76R, Q77E, W101R, L107R; referred to as Equad proteins) were generated by using the Drosophila Schneider S2-cell expression system (ThermoFisher Scientific, https://www.thermofisher.com/us/en/home.html). We inserted into the pMT/BiP/V5-His vector (Invitrogen, https://www.thermofisher.com/us/en/home.html) nucleotides corresponding to the following envelope amino acid sequences: 1–399 of DENV-1 strain Nauru/West Pac/1974, 1–399 of DENV-2 strain 16681, 1–397 of DENV-3 strain Sri Lanka/1266/2000, and 1–399 of DENV-4 strain Dominica/814669/1981. Purifications from the S2-cell culture supernatant were carried out via nickel-affinity and subsequent size-exclusion chromatography, as described (*1*).

ELISA

The IgM ELISA was carried out as described previously (*1*) but with modifications needed to acquire single-antigen measurements using serum dilutions. In brief, the Equad protein of each DENV serotype (300 ng/well) was coated separately overnight on Nunc Maxisorb plates (Invitrogen) in 100 µL coating buffer (15 mM Na_2CO_3, 35 mM $NaHCO_3$, pH 9.6) at 4°C. The plates were washed 3 times with 350 µL/well of phosphate-buffered saline with tween (0.05%) and blocked with 200 µL of 5% nonfat milk powder (blocking solution) for 2 h at room temperature. After washing, human serum samples were serially 2-fold diluted from 1:100 to 1:12,800 in blocking solution and 100 µL/well were added and incubated at room temperature for 1.5 h. After another washing step, the horseradish peroxidase–conjugated secondary rabbit anti–human µ-chain IgM antibody (Dianova, https://www.dianova.com/en/) was diluted 1:7,500

in blocking solution and 100 µL/well were added for 1 h at room temperature. After washing, 100 µL/well of TMB substrate (Biozol, https://www.biozol.de/en/) were added and incubated at room temperature for 30 min. The reaction was stopped with 50 µL 1 M H_2SO_4 and signals were read at 450 nm with background reduction at 520 nm in a micro plate reader (Infinite M200; Tecan, https://www.tecan.com/). All tests were performed in duplicates and in 2 independent experiments.

Appendix Table. Positivity cutoffs for the determination of endpoint titers for DENV serotype–specific IgM ELISA*

Equad protein	Cutoff†
DENV-1	0.2766
DENV-2	0.2410
DENV-3	0.2186
DENV-4	0.2193

*DENV, dengue virus; Equad, envelope protein with 4 amino acid changes.
†Cutoffs were calculated by taking the value of the mean plus 2× the SD of 15 negative control serum samples.

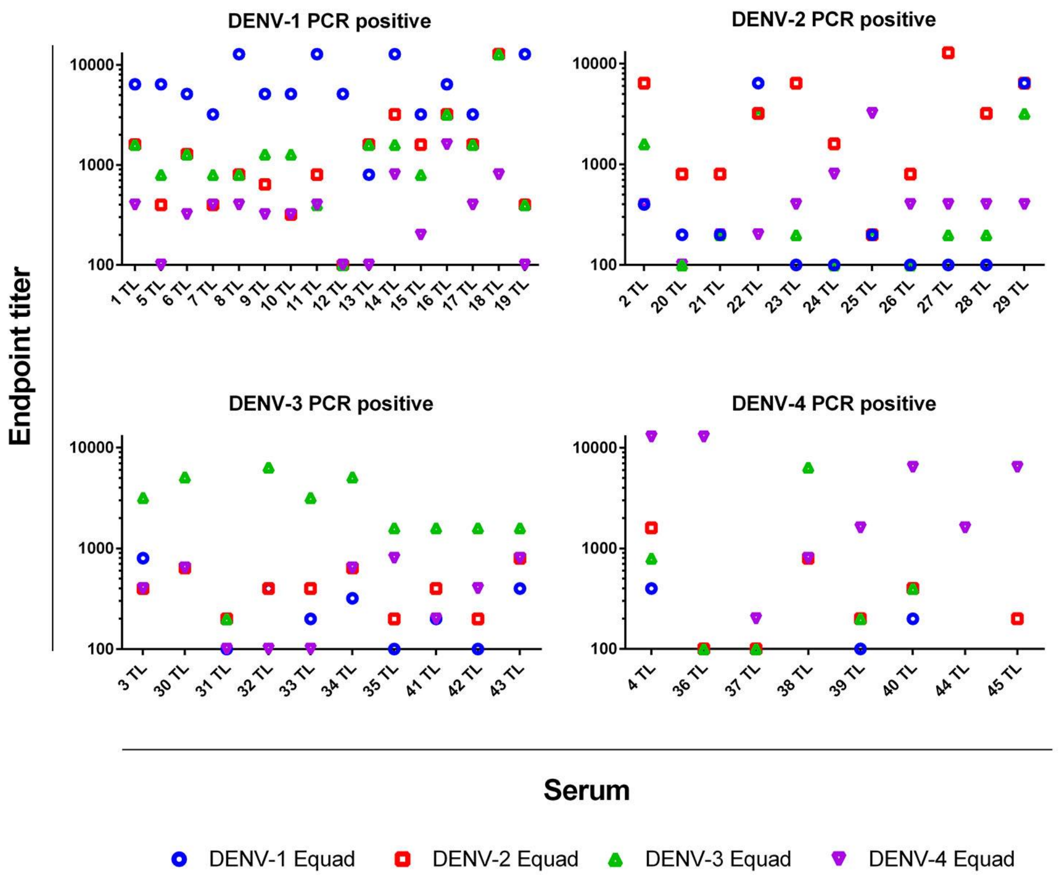

Appendix Figure 1. IgM endpoint titers of DENV-1–4 PCR–positive serum samples from returning travelers, 2013–2016, on DENV 1–4 Equad proteins. Equad proteins are the envelope proteins from DENV with 4 amino acid changes (T76R, Q77E, W101R, and L107R). DENV, dengue virus.

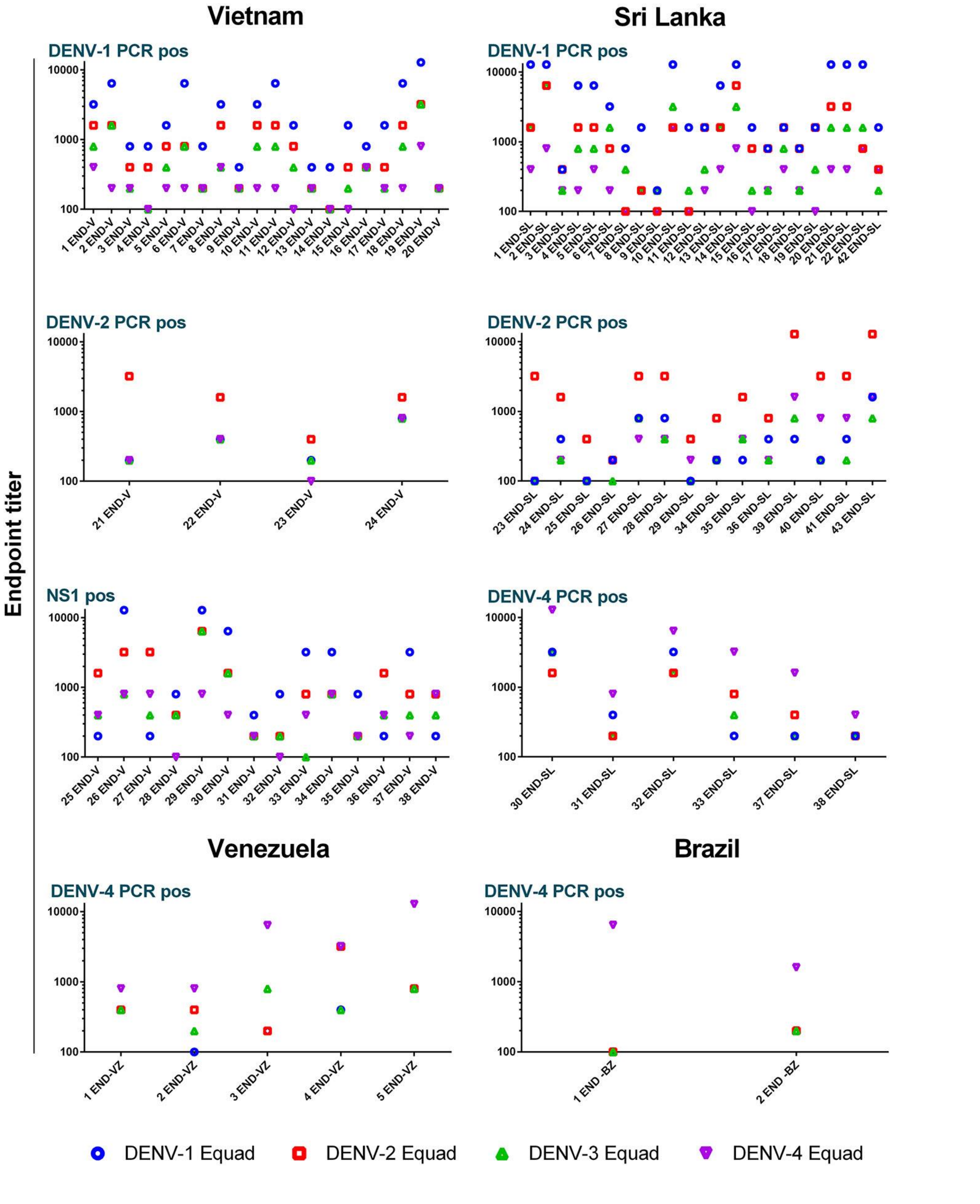

Appendix Figure 2. IgM endpoint titers of DENV-1, DENV-2, and DENV-4 PCR–positive and NS1 antigen–positive serum samples from residents of dengue endemic countries Vietnam, Sri Lanka, Venezuela, and Brazil, 2013–2018, on DENV 1–4 Equad proteins. Equad proteins are the envelope proteins from DENV with 4 amino acid changes (T76R, Q77E, W101R, and L107R). DENV, dengue virus.

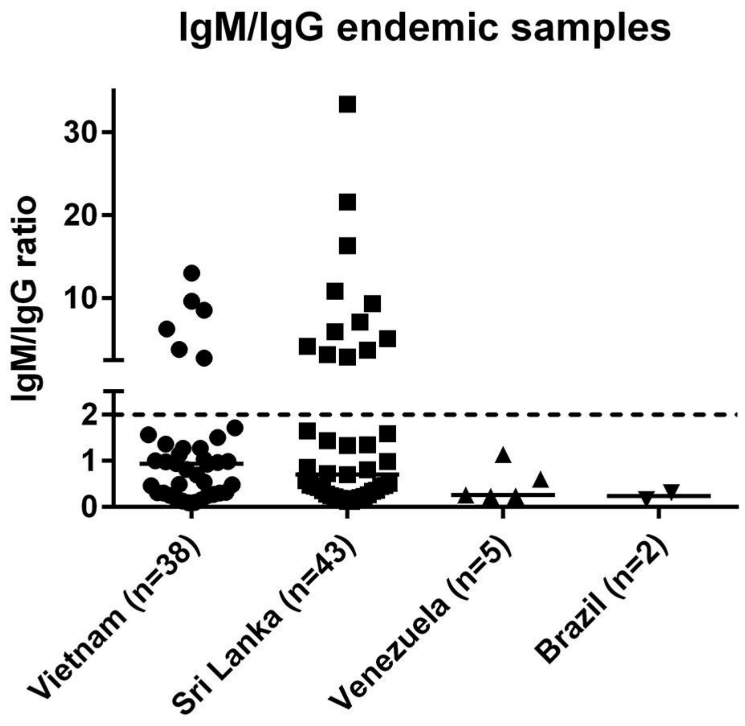

Appendix Figure 3. IgM/IgG ratios for serum samples obtained from patients in the DENV endemic countries of Vietnam (n = 38), Sri Lanka (n = 43), Venezuela (n = 5), and Brazil (n = 2), 2013–2018. The DENV-IgG ELISA was performed as previously described (*1*).The dotted line represents a cutoff for the discrimination of probable primary and secondary infections in which samples with values placed above the line represent primary infections (*2*,*3*).

References

1. Rockstroh A, Barzon L, Pacenti M, Palù G, Niedrig M, Ulbert S. Recombinant envelope-proteins with mutations in the conserved fusion loop allow specific serological diagnosis of dengue-infections. PLoS Negl Trop Dis. 2015;9:e0004218. PubMed http://dx.doi.org/10.1371/journal.pntd.0004218

2. Domingo C, de Ory F, Sanz JC, Reyes N, Gascón J, Wichmann O, et al. Molecular and serologic markers of acute dengue infection in naive and flavivirus-vaccinated travelers. Diagn Microbiol Infect Dis. 2009;65:42–8. PubMed http://dx.doi.org/10.1016/j.diagmicrobio.2009.05.004

3. Carrasco CD. ECDC training workshop on laboratory diagnosis of dengue virus infections, Berlin, 23-27 January 2012. Diagnostics algorithms in dengue infections. 2012 [cited 2018 Apr 7]. http://enivd.de/ECDC/pdf/dc_ml_24_cd_v.pdf
